# Icaritin Inhibits Migration and Invasion of Human Ovarian Cancer Cells *via* the Akt/mTOR Signaling Pathway

**DOI:** 10.3389/fonc.2022.843489

**Published:** 2022-04-01

**Authors:** Lvfen Gao, Yuan Ouyang, Ruobin Li, Xian Zhang, Xuesong Gao, Shaoqiang Lin, Xiaoyu Wang

**Affiliations:** ^1^Department of Obstetrics and Gynecology, The First Affiliated Hospital of Jinan University, Guangzhou, China; ^2^Department of Obstetrics and Gynecology, Guangzhou Panyu Central Hospital, Guangzhou, China; ^3^Integrated Traditional and Western Medicine Research Center, The First Affiliated Hospital of Guangdong Pharmaceutical University, Guangzhou, China

**Keywords:** ovarian cancer, icaritin, drug-resistant, migration, invasion, Akt, mTOR

## Abstract

Ovarian cancer (OC) is the most lethal of all gynecologic malignancies with poor survival rates. Although surgical treatment and chemotherapy had advanced to improve survival, platinum-based chemoresistance remains a major hurdle in the clinical treatment of OC. The search for novel active ingredients for the treatment of drug-resistant OC is urgently needed. Here, we demonstrated that icaritin, the main active ingredient derived from the traditional Chinese herb *Epimedium* genus, significantly suppressed the proliferation, migration, and invasion of both drug-susceptible and cisplatin-resistant OC cells *in vitro*. Mechanistically, icaritin at 20 μM significantly inhibited the phosphorylation of Akt and mTOR, as well as decreased the expression of vimentin and increased the expression of E-cadherin. Our data indicate that icaritin, a prenylated flavonoid natural product, could serve as a potential inhibitor of cisplatin-resistant OC by inhibiting the Akt/mTOR signaling pathway.

## Introduction

Ovarian cancer (OC) is the 5th leading cause of cancer deaths among women in the developed world (1). It remains a challenge to screen or detect OC at the early stages due to non-specific symptoms and lack of reliable biomarkers, resulting in OC being diagnosed at the advanced stage (2). Currently, surgery together with radiotherapy and chemotherapy remains the standard treatment for OC (3). Cisplatin is a front-line chemotherapeutic agent for OC (4). Cisplatin can block DNA transcription by direct covalent binding to nuclear DNA, thus exerting an anticancer activity (5, 6). Unfortunately, the 5-year survival rate is still hovering at about 30% because of cisplatin resistance, which is the major reason for chemotherapy failure. Therefore, to investigate novel target agents to effectively prevent or overcome cisplatin resistance is urgently needed.

Icaritin, a prenylated flavonoid natural product, is commonly recognized as one of the active compounds of the traditional Chinese herb *Epimedium* genus, which has been widely used as a tonic, an aphrodisiac, and an antirheumatic drug in China, Japan, and Korea for thousands of years (7). It has been demonstrated that icaritin possesses a range of different biological and pharmacological functions in non-neoplastic diseases, such as preventing osteoporosis (8) and having cardioprotective (9), neuroprotective (10), and immunoregulatory effects (11). Lately, icaritin and its derivates have attracted great attention in terms of its antitumor effects against various solid tumors including lung cancer (12), prostate cancer (13), hepatocellular carcinoma (14), glioblastoma multiforme (15), and esophageal cancer (16). Moreover, it still exerts promising activity in female tumors, such as breast cancer (17), cervical cancer (18), and also OC in our previous work (19). Icaritin exerts antitumor effects mainly by inhibiting cell proliferation, inducing cell differentiation and apoptosis, suppressing cell migration and invasion, regulating the function of microRNAs, targeting stem cells, and reversing multidrug resistance (12, 13, 16, 20). Numerous signaling pathways are involved in the anticancer activities of icaritin, such as PTEN/Akt, Akt/mTOR, NF-κB, and MAPK/ERK pathways (12, 19, 21). In our previous work, we clarified that the anti-proliferative effects of icaritin on OC may be associated with the activation of p53 and the suppression of the Akt/mTOR pathway. However, the efficacies of icaritin against the migration and invasion of the OC cells and its underlying mechanism have not yet been illuminated.

Here, we sought to investigate the anticancer effect and the underlying mechanism of icaritin on the migration and invasion of human cisplatin-sensitive cells A2780s and the corresponding cisplatin-resistant cells A2780cp. Our results showed that icaritin significantly suppressed the epithelial–mesenchymal transformation (EMT) and migration of both A2780s and A2780cp cells *in vitro* through inhibition of the Akt/mTOR signaling pathway. Our study indicates that icaritin has the potential to inhibit the tumor metastases associated with cisplatin-resistant OC.

## Materials and Methods

### Reagents

Icaritin was a gift from the Laboratory of the Department of Obstetrics and Gynecology, National University Hospital, Yong Loo Lin School (Singapore). RPMI-1640 medium, fetal bovine serum (FBS), and penicillin–streptomycin (PS) were purchased from Gibco (Life Technologies, NY, USA). Matrigel was from BioCoat (Corning, New York, NY, USA). The antibodies against GADPH, p-mTOR, mTOR^2448^, p-Akt^473^, and Akt were from Cell Signaling Technology (Danvers, MA, USA). Cisplatin, MTT (3-(4,5-dimethylthiazol-2-yl)-2,5-diphenyltetrazolium bromide), and other chemicals were obtained from Sigma (St Louis, MO, USA).

### Cells and Cell Culture

The human cisplatin-sensitive OC cell line A2780s and human cisplatin-resistant OC cell line A2780cp were kindly provided by Dr. Benjamin K. Tsang (Ottawa Hospital Research Institute, Ottawa, ON, Canada) (22). These cells were cultured in RPMI-1640 and supplemented with 10% FBS (v/v) and 1% PS (v/v) in a humidified atmosphere of 5% CO_2_ at 37°C. All cell lines were authenticated by STR Multi-amplification Kit and tested negative for mycoplasma using the Mycoplasma Detection Set (M&C Gene Technology, Beijing, China).

### Cell Viability Assays

MTT assay was used to evaluate the cell viability. Cells (1 × 10^4^/well) were seeded in 96-well plates and treated with icaritin at final concentrations of 10–50 μM for 24, 48, and 72 h. MTT (10 μl) solution was added to each well for 4 h after the preset time; then the supernatant was removed. Dimethyl sulfoxide (DMSO) (150 μl) was added to the well for 10 min. The absorbance was measured at 490 nm using a microplate reader (BioTek Synergy HT, Winooski, VT, USA). All experiments were performed three times to determine their reproducibility.

### Wound-Healing Scratch Assay

A2780s and A2780cp cells (5 × 10^6^/well) were seeded in 6-well plates in RPMI-1640 medium containing 10% FBS for 24 h, and the adherent monolayer cells of each plate were scratched at the same size and then treated with icaritin (20 μM) or cisplatin (1.6 μg/ml) for 0, 12, and 24 h. The size at the site of each scratch was recorded by a microscope at 0, 12, and 24 h. The data of the healing condition were calculated by software ImageJ.

### Cell Migration and Invasion Analysis

A2780s and A2780cp cells were firstly cultured in serum-free media condition for 12 h, and the cells were then collected and counted after being exposed to icaritin (20 μM) or cis-platinum (1.6 μg/ml) for 12 h. Cells (1 × 10^4^/well) were cultured in the upper chamber of transwell covered with (invasion) or without (migration) matrigel in the absence of FBS, while RPMI-1640 was added in the bottom well containing 20% FBS as a chemoattract factor. After being cultured for 12 h (for cell migration assay) or 24 h (for cell invasion assay), the migrated or invaded cells were fixed with 3% paraformaldehyde for 20 min and then stained by crystal violet for 10 min. The cells were then randomly photographed and counted.

### Western Blotting

A2780s and A2780cp cells were treated with or without icaritin (10 or 20 μM) for 24 h. Afterward, the cell pellets were collected by centrifugation and rinsed with phosphate-buffered saline (PBS). Whole lysates were prepared with radioimmunoprecipitation assay (RIPA) buffer (1× PBS, 0.1% sodium dodecyl sulfate (SDS), 1% NP-40, and 0.5% sodium deoxycholate) supplemented with protease inhibitor cocktail, 10 mM of β-glycerophosphate, 1 mM of sodium orthovanadate, 10 mM of sodium fluoride, and 1 mM of phenylmethylsulfonyl fluoride; protein samples were subsequently obtained. Bicinchoninic Acid Assay Kit was used to examine the expression of total proteins, and equal amounts of protein were separated on 10% SDS–polyacrylamide gel electrophoresis (PAGE) gels at 125 V for 1.2 h. Transfer to nitrocellulose membranes was performed at 100 V for 1 h. Membranes were blocked using blocking buffer [(bovine serum albumin (BSA)] and then probed with primary antibodies. After incubation with horseradish peroxidase (HRP)-conjugated secondary antibodies in blocking buffer, a chemiluminescence detection system (Thermo Fisher Scientific, Waltham, MA, USA) was used to detect visualized protein bands. GAPDH was used as an internal control. Densitometry was performed using ImageJ software.

### Statistical Analysis

GraphPad Prism 5.0 software was used for statistical analyses (GraphPad Software, Inc., San Diego, CA, USA). All data are presented as the mean values with a standard error of the mean (SEM). Significant differences between the two groups were evaluated using the 2-tailed unpaired *t*-test, and significant differences between more than two groups were evaluated using 1-way ANOVA followed by Tukey’s *post*-*hoc* test. *p* < 0.05 was considered significant.

## Results

### Icaritin Inhibits the Proliferation of Human Ovarian Cancer A2780s and A2780cp Cells

**Figure 1:**
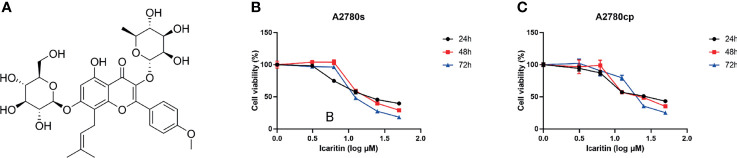
Chemical structure of icaritin and its effects on cell viability in A2780s and A2780cp cells. **(A)** The chemical structure of icaritin. **(B)** A2780s cells were treated with different concentrations (0–50 μM) of icaritin for 24, 48, and 72 h. The cell viability was measured by MTT assay. The data are indicated as means ± SEM of three independent experiments. **(C)** A2780cp cells were treated with different concentrations (0–50 μM) of icaritin for 24, 48, and 72 h. The cell viability was measured by MTT assay. The data are represented as means ± SEM of three independent experiments.

To evaluate the effects of icaritin (**Figure 1A**) on the growth of OC cells, cells were treated with icaritin at different concentrations for 24, 48, and 72 h, and the cell viability was assessed by MTT assay. The results showed that icaritin inhibited both the proliferation of cisplatin-sensitive OC cells A2780s and the cisplatin-resistant OC cells A2780cp in a dose- and time-dependent manner (**Figures 1B, C**). The IC_50_ values of icaritin on A2780s cells were 23.41, 21.42, and 14.9 μM after drug treatment for 24, 48, and 72 h, respectively. And the IC_50_ values of icaritin on A2780cp cells were 28.59, 25, and 22.06 μM after drug treatment for 24, 48, and 72 h, respectively. These data suggest that icaritin inhibits the proliferation of both the cisplatin-sensitive and cisplatin-resistant OC cells.

### Icaritin Suppresses the Wound Healing Ability of A2780s and A2780cp Cells

**Figure 2:**
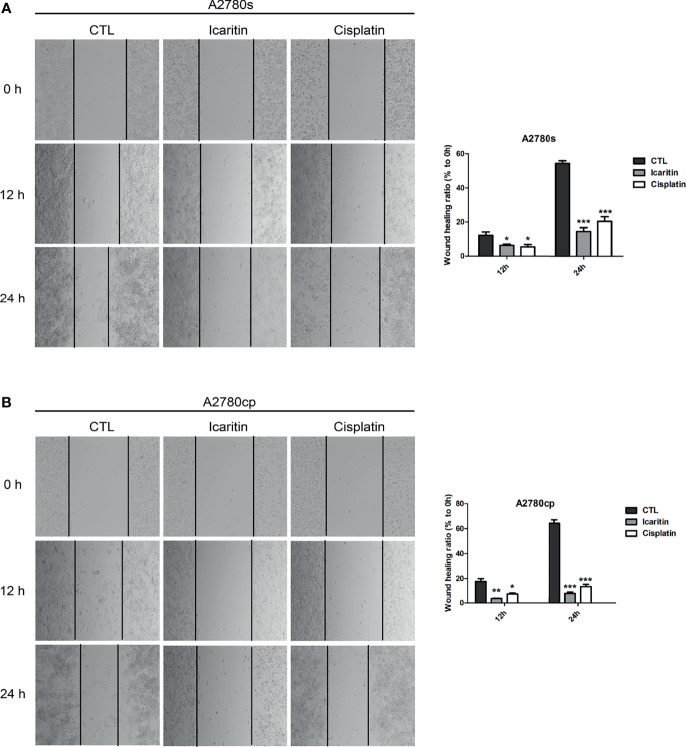
The effects of icaritin or cisplatin on the horizontal motility of A2780s and A2780cp cells by wound-healing scratch assay. **(A)** A2780s were treated with icaritin (20 μM) or cisplatin (1.6 μg/ml) for 12 h or 24 h, respectively. Representative images and quantification of the migrated cells are shown. Data are represented as means ± SEM. ***p < 0.001 by 2-tailed Student’s *t*-test. **(B)** A2780cp cells were treated with icaritin (20 μM) or cisplatin (1.6 μg/ml) for 12 h or 24 h, respectively. Representative images and quantification of the migrated cells are shown. Data are represented as means ± SEM. *p < 0.05, ***p < 0.001 compared to CTL group by 1-way ANOVA followed by Tukey’s *post-hoc* test. **p < 0.01.

To determine whether icaritin affected the wound healing ability of both A2780s and A2780cp cells, we performed the scratch assay. Our results showed that icaritin (20 μM) inhibited the wound healing ability of both A2780s and A2780cp cells in a time-dependent manner (**Figures 2A, B**). The inhibitory rates of icaritin on the wound healing of A2780s were 51.9% and 26.6% at 12 and 24 h as compared to the CTL group, respectively. And the inhibitory rates of icaritin on the wound healing of A2780cp were 19.6% and 12.4% at 12 and 24 h as compared to the CTL group, respectively.

### Icaritin Inhibits the Migration of A2780s and A2780cp Cells

**Figure 3:**
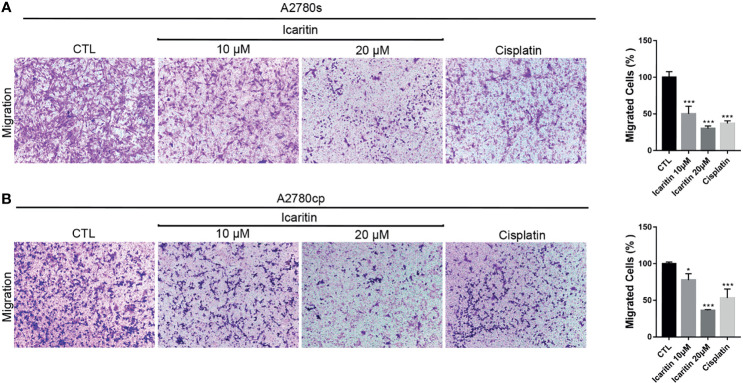
Icaritin suppresses the migration of ovarian cancer (OC) cells. **(A)** A2780s were treated with icaritin (20 μM) or cisplatin (1.6 μg/ml) for 12 h. Representative images and quantification of the migrated cells are shown. Data are represented as means ± SEM. ***p < 0.001 by 2-tailed Student’s *t*-test. **(B)** A2780cp cells were treated with icaritin (20 μM) or cisplatin (1.6 μg/ml) for 12 h. Representative images and quantification of the migrated cells are shown. Data are represented as means ± SEM. *p < 0.05, ***p < 0.001 compared to CTL group by 1-way ANOVA followed by Tukey’s *post-hoc* test.

We next investigated the effect of icaritin in the cell migration of human OC cells. Transwell assays showed that icaritin treatment significantly decreased the number of migrated A2780s and A2780cp cells in a dose-dependent manner, when compared to the control group (**Figures 3A, B**). The inhibitory rates of icaritin on the migration of A2780s were 51.20% and 70.13% at doses of 10 and 20 μM, respectively. And the inhibitory rates of icaritin on the migration of A2780cp were 33.63% and 81.95% at doses of 10 and 20 μM, respectively.

### Icaritin Attenuates the Invasion of A2780s and A2780cp Cells

**Figure 4:**
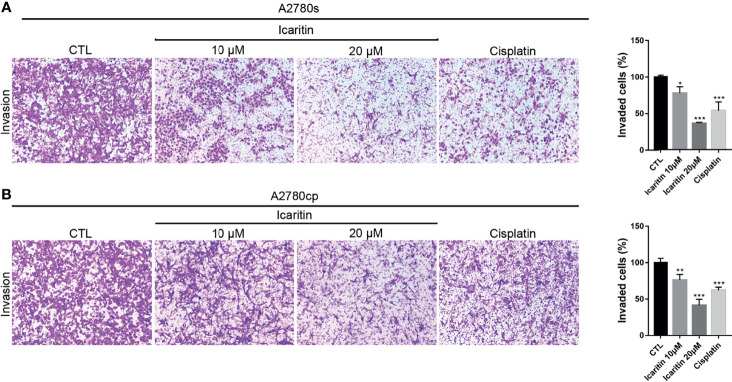
Icaritin inhibits the invasion of ovarian cancer (OC) cells. **(A)** A2780s were treated with icaritin (20 μM) or cisplatin (1.6 μg/ml) for 24 h. Representative images and quantification of the invaded cells are shown. Data are represented as means ± SEM. ***p < 0.001 by 2-tailed Student’s *t*-test. **(B)** A2780cp cells were treated with icaritin (20 μM) or cisplatin (1.6 μg/ml) for 24 h. Representative images and quantification of the invaded cells are shown. Data are represented as means ± SEM. *p < 0.05, **p < 0.01, ***p < 0.001 compared to CTL group by 1-way ANOVA followed by Tukey’s *post-hoc* test.

We utilized matrigel invasion chambers to evaluate the effect of icaritin on the *in vitro* invasion of A2780s and A2780cp cells. Transwell assays showed that icaritin treatment significantly decreased the number of invaded A2780s and A2780cp cells in a dose-dependent manner, when compared to the control group (**Figures 4A, B**). The inhibitory rates of icaritin on the invasion of A2780s were 22.15% and 63.83% at doses of 10 and 20 μM, respectively. And the inhibitory rates of icaritin on the invasion of A2780cp were 22.83% and 58.58% at doses of 10 and 20 μM, respectively.

### Icaritin Inhibits the Akt/mTOR Signaling Pathway in A2780s and A2780cp Cells

**Figure 5:**
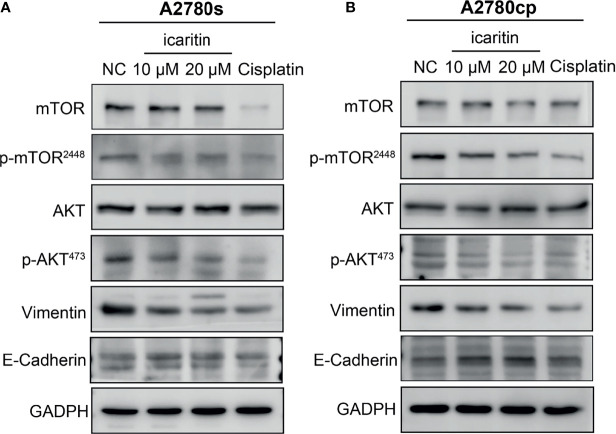
Icaritin inhibits epithelial–mesenchymal transformation (EMT) of ovarian cancer (OC) cells *via* the Akt/mTOR signaling pathways. **(A)** Icaritin decreased the phosphorylation levels of Akt and mTOR, and the expression of vimentin, accompanied by increased expression of E-cadherin in A2780s cells. **(B)** Icaritin decreased the phosphorylation levels of Akt and mTOR, and the expression of vimentin, accompanied by increased expression of E-cadherin in A2780cp cells.

We further evaluated the underlying mechanism of icaritin on inhibition of OC cell migration and invasion. Given that Akt and its important downstream executor mTOR played a critical role in controlling the ability of cell migration and invasion, we investigated whether icaritin can restrain the Akt/mTOR signaling pathway. We found that icaritin significantly suppressed the phosphorylation of Akt and mTOR, but it has a negligible effect on the expression of Akt and mTOR in both the A2780s and A2780cp cells (**Figures 5A, B**). EMT was critical for tumor cell migration, invasion, and tumor metastasis (23); our results showed that icaritin significantly decreased the expression of mesenchymal markers (including N-cadherin and vimentin) and increased expression of epithelial marker E-cadherin in both the A2780s and A2780cp cells (**Figures 5A, B**). Taken together, these data indicate that icaritin inhibited tumor cell motility by suppressing EMT.

## Discussion

Icaritin is the major bioactive component of *Epimedium*, which has been used as Chinese traditional medicine for thousands of years. Many studies demonstrated that numerous traditional Chinese medicine monomers have the effect of reversing tumor resistance (24–26). Extensive evidence showed that icaritin displayed anti-neoplastic activities in a variety of human malignancies both *in vitro* and *in vivo* (13, 27–29). For the first time, our previous study revealed that icaritin induced OC cell apoptosis through activating p53 and suppressing the Akt/mTOR signaling pathway (19). However, the effects of icaritin on migration and invasion of OC cells had not been thoroughly investigated. Therefore, we explored in the present study if icaritin had a significant repressive effect on migration and invasion of both cisplatin-sensitive and cisplatin-resistant OC cells by inhibiting the Akt/mTOR signaling pathway.

It is well established that the migratory and invasive capacity of tumor and stromal cells is linked with tumor metastasis. At present, it is widely believed that the fatal harm of a malignant tumor to the human body mainly lies in tumor metastasis. The migratory phenotype is the precondition for metastatic spreading. Thus, determining the effect of novel agents on migratory and invasive capabilities of tumor cells and clarifying the underlying mechanisms is highly relevant for cancer diagnosis, prognosis, and treatment. It is of great significance to inhibit the motility of tumor cells and prevent the cells from breaking through the basal layer and leaving the primary lesion. A wide variety of assays can be used to assess the migratory or invasion potential and activity of cells *in vitro*, such as wound-healing assay, transwell migration assay, cell exclusion zone assay, transwell assay, 3D cell tracking, and spheroid confrontation assay. In our present experiments, the emphasis was placed on examining the inhibitory activity of icaritin on migratory and invasive motility of OC cells by using wound-healing assay and transwell migration/invasion assay. The results of our study demonstrated that icaritin inhibited the proliferation, migration, and invasion of both A2780s and A2780cp cells in a dose- and time-dependent manner, whose activities were comparable to those of cisplatin. However, for the cisplatin-resistant A2780cp cells, the anti-migration and anti-invasion effects of icaritin were better than those of cisplatin, and it was important to note that cisplatin-resistant OC had the ability to metastasize. Taken together, icaritin is a promising antitumor agent for both cisplatin-sensitive and cisplatin-resistant OC.

Drug resistance is a complicated phenomenon, which has been recognized to severely limit therapeutic outcomes. OC is one of the lethal malignancies in women and cisplatin-based chemotherapy remains the main treatment of OC patients. However, its clinical success is often diminished by chemoresistance. The chemoresistance mechanisms are usually classified into two categories, intrinsic and acquired resistance, but the underlying mechanism of chemoresistance in OC is not completely understood (30). Aberrant activation of the PI3K/Akt/mTOR pathway had been found in various cancers and had been suggested to stimulate proliferation and drug resistance (31). It was reported that the PI3K/Akt/mTOR signaling pathway, EMT, and cancer stem cells played important roles in tumor progression, metastasis, and chemoresistance (32). Numerous studies demonstrated that inhibition of the PI3K/Akt/mTOR signaling pathway alleviated OC chemoresistance (33–35). Icaritin has been shown to have an anticancer effect against various drug-resistance cell types *via* different signaling pathways. For example, icaritin can effectively reverse the multidrug resistance of multiple myeloma cell line KM3/BTZ by decreasing the expression of HSP27 and increasing the expression of Par-4 (36). Icaritin possessed a potential effect on MG-63 doxorubicin-resistant (MG-63/DOX) cells by decreasing the mRNA and protein levels of multidrug resistance protein 1 (MDR1) and multidrug resistance-associated protein 1 (MRP1) and blocking the phosphorylation of STAT3 (20). Icaritin reversed multidrug resistance of HepG2/ADR human hepatoma cells *via* downregulation of MDR1 and P-glycoprotein expression (37). Here, we found that icaritin inhibited the migration and invasion of cisplatin-resistant OC cells. In addition, the Western blotting analysis revealed that icaritin suppressed the Akt/mTOR signaling pathway and process of EMT on both the cisplatin-sensitive and cisplatin-resistant OC cells. Taken together, this study proposes the anticancer mechanism of icaritin in OC.

In conclusion, icaritin could be a potential anti-metastatic agent in cisplatin-resistant OC, and the mechanism might be associated with the inhibition of the Akt/mTOR signaling pathway.

## Data Availability Statement

The raw data supporting the conclusions of this article will be made available by the authors, without undue reservation.

## Author Contributions

LG and YO are considered joint co-first authors for this study. LG, SL, and XW conceived and designed the experiments. YO and RL performed the experiments. XZ and XG analyzed statistical data. LG was a major contributor in writing the manuscript. All authors read and approved the final version of the manuscript.

## Funding

This research was supported by the Medical Scientific Research Foundation of Guangdong Province (no. A2017203) and the Project of Traditional Chinese Medicine in Guangdong (no. 20162045).

## Conflict of Interest

The authors declare that the research was conducted in the absence of any commercial or financial relationships that could be construed as a potential conflict of interest.

## Publisher’s Note

All claims expressed in this article are solely those of the authors and do not necessarily represent those of their affiliated organizations, or those of the publisher, the editors and the reviewers. Any product that may be evaluated in this article, or claim that may be made by its manufacturer, is not guaranteed or endorsed by the publisher.
